# Current patient perspectives of vulvovaginal candidiasis: incidence, symptoms, management and post-treatment outcomes

**DOI:** 10.1186/s12905-019-0748-8

**Published:** 2019-03-29

**Authors:** Junko Yano, Jack D. Sobel, Paul Nyirjesy, Ryan Sobel, Valerie L. Williams, Qingzhao Yu, Mairi C. Noverr, Paul L. Fidel

**Affiliations:** 1 Center of Excellence in Oral and Craniofacial Biology, School of Dentistry, Louisiana State University Health Sciences Center, New Orleans, LA USA; 2 Biostatistics Program, School of Public Health, Louisiana State University Health Sciences Center, New Orleans, LA USA; 3 Department of Obstetrics and Gynecology, School of Medicine, Louisiana State University Health Sciences Center, New Orleans, LA USA; 4 Department of Internal Medicine, Wayne State University, School of Medicine, Detroit, MI USA; 5 Department of Obstetrics and Gynecology, Drexel University College of Medicine, Philadelphia, PA USA; 6 Thomas Jefferson University Hospital, Sydney Kimmel Medical College, Philadelphia, PA USA; 7 Present Address: Department of Microbiology and Immunology, School of Medicine, Tulane University, 1430 Tulane Ave, New Orleans, LA USA

**Keywords:** Vaginitis, Vulvovaginal candidiasis, VVC, RVVC, Epidemiology, Incidence rates, *Candida albicans*, Symptomatology, Risk factors, Disease management

## Abstract

**Background:**

Vulvovaginal candidiasis (VVC) is a common infection affecting women worldwide. Reports of patterns/risk factors/trends for episodic/recurrent VVC (RVVC) are largely outdated. The purpose of this study was to obtain current patient perspectives of several aspects of VVC/RVVC.

**Methods:**

Business cards containing on-line survey information were distributed to healthy volunteers and patients seeking standard, elective, or referral gynecologic care in university-affiliated Obstetrics/Gynecology clinics. The internet-based questionnaire was completed by 284 non-pregnant women (78% Caucasian, 14% African American, 8% Asian).

**Results:**

The majority of the participants (78%) indicated a history of VVC with 34% defined as having RVVC. The most common signs/symptoms experienced were itching, burning and redness with similar ranking of symptoms among VVC and RVVC patients. Among risk factors, antibiotic use ranked highest followed by intercourse, humid weather and use of feminine hygiene products. A high number of respondents noted ‘no known cause’ (idiopathic episodes) that was surprisingly similar among women with a history of either VVC or RVVC. VVC/RVVC episodes reported were primarily physician-diagnosed (73%) with the remainder mostly reporting self-diagnosis and treating with over-the-counter (OTC) medications. Most physician-diagnosed attacks utilized a combination of pelvic examination and laboratory tests followed by prescribed antifungals. Physician-treated cases achieved a higher level of symptom relief (84%) compared to those who self-medicated (57%). The majority of women with RVVC (71%) required continual or long-term antifungal medication as maintenance therapy to control symptoms.

**Conclusions:**

Current patient perspectives closely reflect historically documented estimates of VVC/RVVC prevalence and trends regarding symptomatology, disease management and post-treatment outcomes.

**Electronic supplementary material:**

The online version of this article (10.1186/s12905-019-0748-8) contains supplementary material, which is available to authorized users.

## Background

Vulvovaginal candidiasis (VVC) is a common fungal infection caused by *Candida* species, predominantly *C. albicans* [1]. Historical reports approximate that 70% of all women will have at least one episode of VVC during their reproductive years [2]. The pathological hallmark of the disease is an acute inflammatory condition of the vulva and vaginal mucosa induced by and accompanied with overgrowth of *Candida* organisms that normally exists as a quiescent vaginal commensal [3]. Signs/symptoms of VVC are typically characterized by white clumpy discharge, burning, redness and itching in the vulva and vagina, and dyspareunia [4]. The onset of most VVC cases is believed to be associated with a wide range of predisposing factors or triggering events including the use of antibiotics, increased estrogen levels (e.g. high estrogen oral contraceptives, hormone replacement therapies, pregnancy), uncontrolled diabetes mellitus, sexual activities and tight-fit clothing [2, 5]. In addition, an estimated 8 to 10% of women are susceptible to recurrent VVC (RVVC), having 4 or more episodes per annum [4]. Multiple recurring infections are often idiopathic without regard to the array of potential risk factors. Unlike most episodic or sporadic VVC, RVVC cases require maintenance regimens with long-term use of antifungals over several months or longer to avoid recurrence [4, 6].

Despite the high incidence rates worldwide, epidemiological data supporting the current estimates of VVC or RVVC prevalence rates had been limited, largely historical, and often anecdotal. More recently, however, several global studies have been reported. One reports the worldwide prevalence of RVVC at approximately 138 million women annually, and an additional 372 million over one’s lifetime, causing substantial morbidity and economic burden [7]. Another large-scale multi-country internet panel survey indicated a lifetime RVVC prevalence rate of 9% by age 50 with the vast majority of episodes occurring between 19 to 35 years of age [8], which was remarkably similar to the historical estimates [9]. Despite these global studies on incidence/prevalence rates worldwide, current information/perspectives on VVC/RVVC disease trends is still needed and critical for reporting on aspects of vaginal infections, including diagnostics and therapeutic approaches, understanding host immunity and pathogenesis, and behavioral factors associated with disease etiology. Indeed, a large body of current literature on host defense mechanisms against VVC/RVVC and associated immunopathology [10–14] has relied on long past historical epidemiological citations for VVC/RVVC that may not accurately reflect current disease-associated trends.

The objective of this study was to conduct a contemporary survey of women with a history of VVC and RVVC among general populations and those seeking standard or elective care at several university-affiliated Obstetrics and Gynecology clinics or referral clinics, to determine current trends including diagnostic/management parameters, ranking of disease symptomatology, and post-treatment outcomes, in addition to prevalence rates.

## Methods

### Study designs

The survey included responses by 284 non-pregnant women over a period of February 2016 to May 2018. Business cards containing on-line survey information were distributed to subjects who sought gynecologic care in university-affiliated Obstetrics and Gynecology clinics at the time of visit with a health care provider or via group encounters (meetings, classes, community activities). The university-affiliated Ob/Gyn clinics included the following: Detroit Medical Center, Drexel University, Louisiana State University Health Sciences Center (LSUHSC) – New Orleans and Thomas Jefferson University. Eligible subjects (any adult women) participated in the survey on a voluntary basis by accessing a specified URL via internet and answering a questionnaire using Survey Monkey (http://www.surveymonkey.com). Access to the survey web site was open to the general public without password restriction. The questionnaire was approved by the Institutional Review Board at LSUHSC, New Orleans with a waiver of informed consent. The survey was administered anonymously and included no identifiers linking data to individual respondents.

### Survey contents and interpretations

The survey questionnaire included a total of 13 questions regarding i) participant demographics, ii) previous history of VVC, iii) lifetime/annual frequencies of VVC episodes, iv) vulvovaginal signs and symptoms, v) known causes if any, vi) choice of clinical interventions for diagnoses and treatment regimens, and vii) post-treatment outcomes (Table 1). The questions included 2 to 12 answer choices per question. The participants were presented with the questions one at a time on their device of choice and given the option of ‘no response’. The exception was the first question in which participants answering ‘no history’ were led out of the remainder of the survey. For those questions with more than two answer choices, the responses were combined into two categories for analyses. Not applicable (N/A) answers were interpreted as missing values (Additional file 1).

**Table 1:** List of questions and responses in the survey questionnaire

Questions	Response choices
Demographics	Race
	Ethnicity
	Age
Previous history of VVC/RVVC	Yes
	No
Frequency (lifetime)	1–10 episodes
	> 10 episodes
Frequency (annual)	0–3 episodes
	> 3 episodes
Signs/symptoms^a^	Itching, burning, cottage cheese-like discharge, redness in the vaginal area, vaginal pain, vaginal dryness, vaginal pain, pain during intercourse
Causes^a^	No known cause, oral contraceptives, antibiotics, hormone replacement therapy, diabetes, humid weather, pregnancy, after intercourse, after oral sex, a new sexual partner, feminine hygiene products, other
Diagnosis	Physician-diagnosed with exam and lab test, treated with prescription oral or topical medication.
	Physician-diagnosed with exam and lab test, treated with OTC topical medication.
	Physician-diagnosed with exam only, treated with prescription oral or topical medication.
	Physician-diagnosed with exam only, treated with OTC topical medication.
	Self-diagnosed and treated with OTC topical medication.
	Other.
Relief	Physician-treated, relief
	Self-treated, relief
	Physician-treated, no relief
	Self-treated, no relief
Post-treatment outcome	Cured
	Recurred/relapse
RVVC management	Constant antifungal medication, relief
	Constant antifungal medication, no relief
	As needed antifungal medication, relief
	Avoiding known risk factors without medication

^a^Respondents indicated all applicable choices

### Statistical analyses

Fisher’s exact test was used for a binomial proportion comparing two populations of women choosing one of two responses (i.e. “yes” versus “no”) of the question. For questions with multiple answer choices, the entire population of the respondents were categorized into two groups based on criteria specified for each survey data set and analyzed for binomial distributions. For proportions of RVVC patient age ranges, Pearson’s Chi-squared tests were used to analyze for equal distributions in each age group. All statistical analyses were performed using the SAS version 9.4 software (SAS Institute, Cary, NC).

## Results

### Demographic characteristics of the participants

Race and age group distributions of the survey participants were assessed prior to collecting clinical information in regards to VVC/RVVC. Racial data on respondents who completed the survey included Caucasian (*n* = 202, 77.7%), African American (*n* = 37, 14.2%) and Asian (*n* = 21, 8.1%) women (Table 2). No participants of native American or native Hawaiian/Pacific Islander origins were reported. A small proportion of women identified themselves as Hispanic or Latino (*n* = 20, 8.2%). Approximately one half of the participants were within reproductive age (18–25 years of age, *n* = 36, 13.6%; 26–40 years of age, *n* = 131, 49.4%), and the rest were within perimenopausal or postmenopausal age (41–55 years of age, *n* = 59, 22.3%; 56 or older, *n* = 39, 14.7%).

**Table 2:** Demographics of study participants with a history of VVC/RVVC

Race	Caucasian	77.7% (202)^b^
	African American	14.2% (37)
	Asian	8.1% (21)
	Native American	0.0% (0)
	Native Hawaiian/Pacific Islander	0.0% (0)
Ethnic category	Hispanic or Latino	8.2% (20)
	Not Hispanic or Latino	91.8% (224)
Age	18–25	13.6% (36)
	26–40	49.4% (131)
	41–55	22.3% (59)
	> 55	14.7% (39)
Lifetime history of infection	No history	22.5% (64)
	≥1	77.5% (220)
Annual frequency of infection	≤3	65.4% (132)
	> 3 (RVVC)^a^	34.6% (70)

^a^RVVC, recurrent vulvovaginal candidiasis defined as 4 or more acute episodes in a 12-month period

^b^Values in parentheses indicate the number of respondents who selected each response choice

### Prevalence of VVC/RVVC

**Fig. 1:**
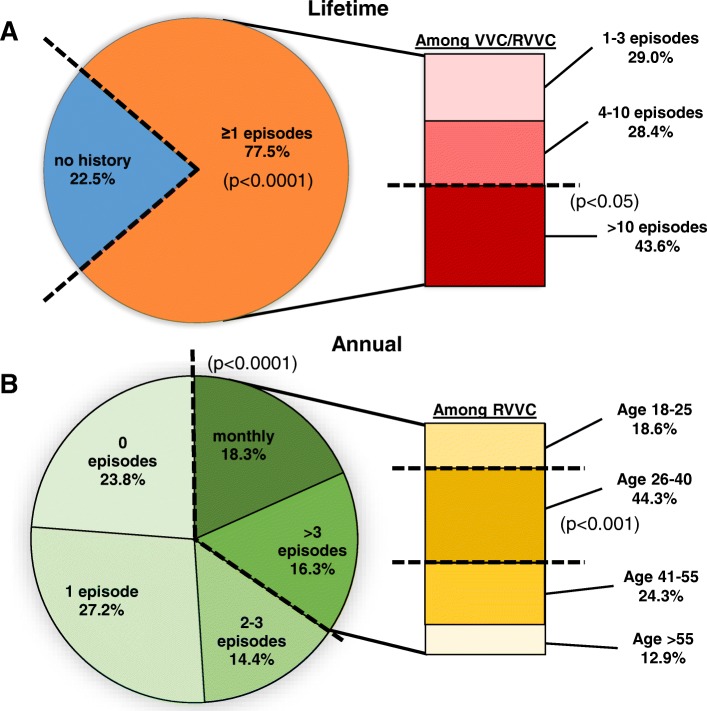
Prevalence of VVC/RVVC and distribution of lifetime/annual frequencies of infection. **a** Lifetime history of VVC in participating women (*n* = 284, pie chart) was assessed by a self-reported survey. Total VVC episodes in respondents with disease history were further stratified by lifetime frequencies (*n* = 204, bar chart). **b** Respondents with previous VVC episodes were classified by annual frequencies (*n* = 202, pie chart), and those with annual frequencies of > 3 VVC episodes were further stratified by age (*n* = 70, bar chart). The percentage in each section indicates the proportion of women among those who reported answers to each parameter. Data were analyzed by Fisher’s exact test for binomial proportions comparing two populations of women categorized by the dashed lines. NS, not significant

Prevalence was determined based on the number of individual VVC/RVVC episodes in a respondent’s lifetime and, if any, annually as well. A significantly large proportion of the respondents (*n* = 220, 77.5%, *p* < 0.0001) reported at least one episode of VVC in their lifetime (Table 2, Fig. 1a). Among those with previous episode(s) of infection, a lower number of respondents reported having > 10 lifetime episodes (*n* = 89, 43.6%) compared to those that reported fewer than 10 lifetime episodes (*n* = 115, 56.4%, *p* < 0.05) (Fig. 1a). Likewise, when evaluated based on the number of VVC episodes per year, the distribution of women with > 3 episodes (i.e. RVVC) (*n* = 70 women, 34.7%,) were significantly lower compared to those with ≤3 annual episodes (*n* = 132, 65.3%, *p* < 0.0001) (Table 2, Fig. 1b). Subsequently, the RVVC population of the respondents were further stratified into specific age groups. The proportion of RVVC women in the range of 26 to 40 years of age was significantly higher than other age groups ranging 18 to 25 years, 41 to 55 years or, > 55 years (*p* < 0.001) (Fig. 1b).

### Clinical features of VVC/RVVC

Information regarding vaginal signs and symptoms experienced during VVC episodes was reported by both women with infrequent history of infection as well as those with a history of recurrence. Overall, the most common clinical characteristics were associated with vaginal inflammation, namely itching (91.2% of all respondents; VVC-92.4%; RVVC-90.0%) followed by burning (68.3% of all respondents; VVC-62.1%; RVVC-81.4%) (Table 3). Additional common features of symptomatic VVC/RVVC episodes included redness (58.1%), vaginal discharge described as thick, white or cottage cheese-like (55.6%), pain during intercourse (40.5%), vaginal pain (38.1%) and vaginal dryness (29.3%). Greater than 50% of the respondents (55.4%) reported that their VVC episodes had no known cause. These idiopathic attacks were reported by similar proportions of women with a history of VVC (51.2%) and RVVC (62.9%). If a cause was reported, antibiotic use was the most common risk factor (37.8%) followed by intercourse (21.6%), hormone-induced conditions (pregnancy, use of oral contraceptives, hormone replacement therapy, 13.7%) and humid weather (11.3%). There was moderate association of VVC/RVVC incidence with use of feminine hygiene products (10.8%), having a new sexual partner (8.3%) or receptive oral sex (6.9%). A small number of cases (< 3.0%) were reported as diabetes-related.

**Table 3:** Clinical features of VVC symptomatology and risk factors associated with disease

Signs/symptoms	Itching	91.2% (187)^a^
	Burning	68.3% (140)
	Redness in the vaginal area	58.1% (119)
	Cottage cheese like discharge	55.6% (114)
	Pain during sex	40.5% (83)
	Vaginal pain	38.1% (78)
	Vaginal dryness	29.3% (60)
Causes	No known cause	55.4% (113)
	Antibiotics	37.8% (77)
	After intercourse	21.6% (44)
	Humid weather	11.3% (23)
	Use of feminine hygiene product or douching	10.8% (22)
	Having a new sexual partner	8.3% (17)
	Pregnancy	7.8% (16)
	After oral sex	6.9% (14)
	Taking oral contraceptives	5.4% (11)
	Diabetes	2.5% (5)
	Hormone replacement therapy	0.5% (1)
	Others^b^	17.2% (35)

^a^Values in parentheses indicate the number of respondents who selected each response choice

^b^Including high sugar diet, exercising, stress, before/after menstruation and swimming

### Diagnosis and management of VVC/RVVC

**Fig. 2:**
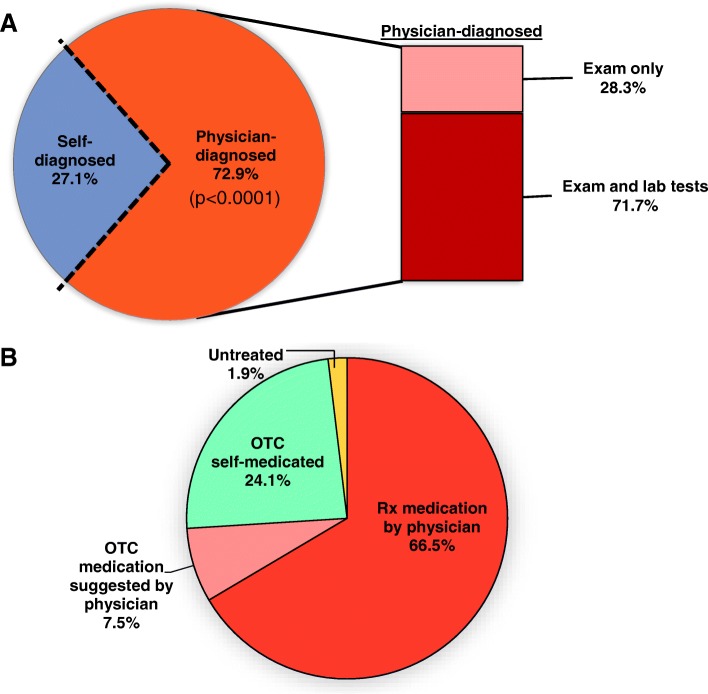
Methods of disease diagnoses and management in women seeking treatment for vaginitis. **a** The process of diagnosing vaginitis conditions by participating women (*n* = 214, pie chart) was assessed by a self-reporting survey. Methods of physician-based diagnoses used in respondents seeking medical care were further classified (*n* = 152, bar chart). **b** The respondents who underwent antifungal treatment (*n* = 212) were categorized based on diagnostic and therapeutic approaches. The percentage in each section indicates the proportion of women among those who reported answers to each parameter. Data (A, pie chart) were analyzed by Fisher’s exact test for a binomial proportion comparing two populations of women opting for physician-based diagnosis and self-diagnosis/other (dashed lines). OCT, over-the-counter (non-prescription)

VVC was either self-diagnosed based on vaginal symptoms alone followed by self-medication with over-the-counter (OTC) antifungals, or diagnosed and treated by a physician. Most respondents in this study had infections diagnosed by physicians (72.9%), with a smaller proportion (27.1%) opting self-diagnosis without seeking medical care (Fig. 2a) (*p* < 0.0001). Among women reporting physician-diagnosed VVC/RVVC, the clear majority of cases (71.7%) were determined by the combination of performing a pelvic examination for signs of vaginitis and conducting laboratory tests involving wet mount microscopy, cultures, or nucleic acid amplification-based detection to confirm the presence of fungal organisms. Of note, several women reported that physician diagnosis was based upon a pelvic examination alone (28.3%). Following physician diagnosis, most VVC cases involved treatment with oral or topical antifungal medications prescribed by the physician (66.5%) with a minor portion instructed by physicians to treat with OTC medications (7.5%) (Fig. 2b). Women who reported self-diagnosed VVC (24.1%) tended to OTC antifungal medications for treatment or occasionally experienced spontaneous clearance of symptoms without treatment (1.9%).

### Clinical outcomes following treatment

Clinical outcomes of women receiving treatment for VVC/RVVC included a spectrum of symptom control or relief to recurrence of symptoms (often rapid). A significantly larger proportion of women who were treated by physicians achieved relief of symptoms (84.4% vs. 15.6% non-relief, *p* < 0.0001) (Table 4). Conversely, the relief rate was considerably lower in women with a history of self-diagnosis and self-directed therapy (57.4% vs. 42.6% non-relief, *p* = 0.06). Among those who attained symptom relief following treatment with an antifungal, proportions of women who ultimately experienced a repeat episode and those who reported no further episodes were similar (53.2% recurrence vs. 46.8% cure, *p* = 0.20). In the RVVC population, 71% achieved symptom relief by utilizing maintenance antifungal therapy or on an as-needed basis, whereas 19% failed to achieve relief of symptoms despite multiple regimens of antifungal medication. A small proportion of RVVC women (9.6%) reported that avoiding triggering factors or events that they recognized as provoking a symptomatic episode was effective management of RVVC.

**Table 4:** Cure rates following treatment

			*p*-value^b^
Physician-treated	Relief	84.4% (141)^a^	*p* < 0.0001
	No relief	15.6% (26)	
Self-medicated	Relief	57.4% (74)	NS^c^
	No relief	42.6% (55)	
Post-treatment outcome	Cure	46.8% (94)	NS^c^
	Recurrence	53.2% (107)	
RVVC^d^ maintenance regimens	Constant/as needed antifungal medication – relief	71.1% (74)	*p* < 0.0001
	Constant antifungal medication – no relief	19.2% (20)	
	Avoiding known risk factors without medication	9.6% (10)	

^a^Values in parentheses indicate the number of respondents who selected each response choice

^b^Data were analyzed by Fisher’s exact test for binomial distribution

^c^NS, not significant

^d^RVVC, recurrent vulvovaginal candidiasis defined as 4 or more acute episodes in a 12-month period

## Discussion

Despite decades of research and high global prevalence, VVC and RVVC continue to present major health issues in affected women [2, 7]. Until very recently, general epidemiological information has been limited in contemporary literature and consequently, reports of current research in a variety of areas related to VVC/RVVC have referenced predominantly long past historical epidemiological data/observations. Results from the current survey provide a current patient perspective for several aspects of VVC/RVVC that can be used to compare to historical data and provide a contemporary resource for citation.

Data collected from 284 women showed a high proportion with a history of at least one previous episode of VVC (78%), with the majority of women with 3 or less annual episodes (65%) and a smaller proportion of those with 4 or more annual episodes (35%, i.e., RVVC). If one removes the RVVC sub-population, the lifetime prevalence is reduced to 68%. In either case, the reported lifetime history of VVC in this population of women is remarkably similar to historical reports (~ 75%) [15–18]. Although the proportion of the RVVC group in this population was relatively high, owing to the inclusion of many women seeking specialist gynecologic care, this provided a large sample size of women with a history of VVC/RVVC (*n* = 204) to provide their perspectives. The race distribution (78% Caucasian, 14% African American, and 8% Asian) was reflective of the participant pool and not designed for matching by age or race.

The current survey estimates that approximately 38% of VVC cases are associated with antibiotic use, similar to previous estimates of 33% [9]. Interestingly, the high rate of responses (55%) denoting ‘no known cause’ for their episodes (idiopathic) was not dominated by RVVC respondents who are characteristically in the idiopathic category relative to cause [4]. Quite the contrary, there was a similar percentage of women with VVC and RVVC noting ‘no known cause’ (52 vs. 63%, respectively). This suggests that while the cause of episodic VVC is often known, there is an equally high proportion of acute VVC cases that are idiopathic similar to RVVC.

As historically reported, signs/symptoms associated with vulvovaginal inflammation (i.e. pruritus, burning, redness) and vaginal discharge are the hallmark manifestations of an acute VVC episode [19], which was also clearly indicated in the current survey. Interestingly, ranking of symptoms reported by those with episodic VVC was comparable to those experienced during RVVC when analyzed independently. Based on the sufficient numbers of women with VVC and RVVC in the survey, these data have significant power to suggest that the clinical pathology of RVVC is quite similar, if not identical, to acute VVC. It is noteworthy, however, that the frequency of women reporting multiple symptoms was higher in those with RVVC. This may be due simply to the relative frequency of the infection and a higher level of attentiveness to the accompanying signs/symptoms.

Among the RVVC population, the highest proportion were in the age ranging from 26 to 40. This is consistent with historical data and likely reflects the reported hormonal and behavioral predisposing factors to infection [20]. The relatively high proportions of RVVC rates in the perimenopausal (41 to 55 years of age) and postmenopausal (56 or older) populations may reflect potential age-related health conditions such as exogenous estrogen use to treat atrophy. Indeed, a study conducted in patients attending a vulvar disease referral clinic reported that nearly 50% of women diagnosed with VVC/RVVC were on hormone replacement therapy [21].

Regarding diagnosis and treatment, results showed that a significantly greater proportion of respondents had their episodes physician-diagnosed and treated with prescription antifungal drugs, leading to control/relief of symptoms in 84% of cases. These observations closely parallel previously reported estimates of 80 to 90% cure rates (defined by resolution of signs/symptoms and negative mycological tests) by treatment with topical or oral azoles [22, 23]. Also, consistent with a previous report estimating that only 38% of cases can be diagnosed correctly by symptomatology alone [19], our survey showed poor relief rate (57%) among women who reported they self-diagnosed and self-medicated with OTC drugs. For RVVC, a previous study reported that relapse occurred in 50% of women who failed to initiate a maintenance regimen [24]. This was reflective in the current survey where > 71% of women with RVVC reported requiring constant/as needed antifungal therapy to sustain relief. The overall relapse rate noted by the respondents (53%) is likely reflective of the relatively large RVVC population surveyed.

We acknowledge several limitations to the current study. It is important to note that the VVC diagnosis/cure data described was solely based on self-reported data as no formal chart screening was performed. As a result, we acknowledge that the open survey format leaves room for inaccuracies that should not be overlooked. For example, poor relief rate among women relying on self-diagnosis may have resulted from self-misdiagnosis. Indeed, signs and symptoms of RVVC are frequently mistaken for many other conditions such as bacterial vaginosis or persistent vulvar vestibulitis, and inadequate diagnosis without clinical examinations often leads to chronic vulvovaginal conditions [7, 25]. But despite potential inaccuracies, these current data are remarkably similar to historical information [19, 22, 23]. The relatively small sample size (*n* = 284) represented another limitation, especially considering the recent internet-based multi-country survey on RVVC prevalence that included 6100 respondents [26]. However, most clinic-based VVC/RVVC studies consist of sample sizes typical of the present study [3, 27–32]. More importantly, however, is the recognition of selection/accrual bias in the current dataset owing to the fact that the participants were mainly comprised of women seeking care or continued care by a gynecologist, and often those specializing in chronic/recurrent VVC. Indeed, > 34% of the respondents reported RVVC. This introduced a significant bias in the participant pool that likely resulted in overestimation of the number of physician-diagnosed VVC/RVVC cases although the overall lifetime VVC incidence rate (~ 78%) were comparable to historical estimates (~ 75%) [15–18]. It is assumed that the proportion of self-diagnosed VVC/RVVC cases would be higher in a more randomized population with all respondents devoid of attending established clinics. We recognize that these results may not be fully representative of a broader general population and thus may be another potential limitation. On the other hand, the inclusion of women with higher than average incidence rates of RVVC may actually be considered advantageous as it provided the opportunity to collect more relevant and informative data with respect to symptomatology, diagnosis, treatment and post-treatment outcomes.

In summary, we provide a contemporary patient perspective of several aspects of VVC and RVVC that while largely confirm historical reports, also include some new insights. Taking into account the overall similarity to historical reports, it bears noting that there has not been any substantial reduction in lifetime or annual prevalence rates of VVC over the past 30+ years despite a number of new drugs and effective maintenance therapy for RVVC. In fact, a study by Denning, et al. predicts an upward trend in RVVC cases by 2030 [7]. This is also despite a plethora of data regarding the pathogenesis of disease and potential immunotherapeutic strategies. Hence, continued research is still needed to impact the incidence of VVC/RVVC. On the positive side, the fact that VVC and RVVC appear to manifest a similar, if not identical, clinical pathology suggests that any diagnostic or immunotherapeutic advances will benefit either condition. Though we recognize the limitations of this study and selection bias of the respondents, these data represent a current set of patient trends in several areas that can serve as a useful resource in future research/publications involving VVC/RVVC.

## Conclusions

A contemporary survey of women with a history of VVC and RVVC provides current trends in prevalence rates, ranking of disease symptomatology, diagnostic/management parameters, and post-treatment outcomes. These current patient perspectives closely reflect previously documented estimates and validate historical reports.

## Additional file

Additional file 1: Participants’ responses to the questionnaire. The participants (*n* = 284) were presented with the survey questionnaire items (Q) regarding previous history of VVC (Q1), lifetime/annual frequencies of VVC episodes (Q2-Q3), vulvovaginal signs and symptoms (Q4), known causes (Q5), choice of clinical interventions for diagnoses and treatment regimens (Q6), post-treatment outcomes (Q7-Q10) and participant demographics (Q11-Q13). A set of 2 to 12 answer choices was included per question. All answer(s) selected by each respondent are indicated (Sheet 1. Raw data). Respondents selecting other as an answer choice in Q5-Q6 provided their specified responses as listed in the corresponding spreadsheets (Sheets 2–3. Q5 others and Q6 others). (XLSX 34 kb)

## Abbreviations

OTC: over the counter
RVVC: recurrent vulvovaginal candidiasis
VVC: vulvovaginal candidiasis
